# Health care professionals’ perspective on children’s participation in health care situations: encounters in mutuality and alienation

**DOI:** 10.1080/17482631.2018.1555421

**Published:** 2018-12-20

**Authors:** Maria Harder, Maja Söderbäck, Albertine Ranheim

**Affiliations:** a School of Health, Care and Social Welfare, Mälardalen University, Västerås, Sweden; b Department of Nursing, Institute of Neurobiology, Care and Society Karolinska Instiutet, Stockholm, Sweden

**Keywords:** Children, encounters, health care professional, health care situations, participation, phenomenological-hermeneutical approach

## Abstract

**Purpose**: Encounters between health care professionals, parents and children in health care services for children are complex as these encounters involve the various perspective and understanding of each person involved. The aim of the study is to describe health care professionals’ understanding of significant encounters with children and parents to uncover the meaning of participation.

**Method**: A qualitative descriptive design was applied. The health care professionals’ narratives (n = 35) of their significant encounters with children were interpreted from the perspective of participation. A phenomenological-hermeneutical approach was used in the analysis.

**Results**: The findings show children’s participation as a dynamic movement in mutuality and alienation which can vary within a situation or between different situations involving the same persons. The movement can occur in mutuality and or in alienation depending on what or towards whom the persons direct themselves. Understanding participation as a movement in health care situations is useful in supporting children’s opportunities to participate from their own perspective and deal with health care examinations.

**Conclusion**: The outcome of a situation can never be predicted. Still, professionals can be aware of their actions in encounters with children.

## Introduction

In health care situations with children, the responsibility of obtaining participation lies on the adults, the professional and the accompanying parent or guardian, but foremost on the professionals. This responsibility is set out by legislation and conventions. Children’s right of participation is clarified in The Swedish Patient Act (SFS, 2014:821). The act proclaims that each child’s perspective regarding examinations and treatments needs to be considered in relation to age and maturity. Furthermore, the Convention on the Rights of the Child (United Nations, 1989) emphasizes children’s rights to make their voice heard in matters that concern them. To fulfil these regulations professionals in health care situations need to be aware of and reflect on their encounters with children from the perspective of participation i.e., the child’s opportunity to take part and contribute in a situation (Söderbäck, Coyne, & Harder, 2011). How an individual contributes, and with what autonomy, is given on individual basis (James & James, 2008). To consider children’s participation, professionals need to be able to distinguish between their own child perspective and the child’s perspective in each situation. Professionals’ child perspective is their view on children’s conditions, experiences and understandings, which will differ from the child’s perspective on their conditions, experiences and understandings (Sommer, Pramling, & Hundeide, 2010).

According to previous research, professionals have difficulties in promoting children’s participation (Coyne, 2006a). Professionals may agree on the importance of considering the child’s perspective and right of being respected. Still, children are positioned as passive onlookers or as passive participants. A child being a passive onlooker means the parent is the one the professional communicates with (Lambert, Glacken, & McCarron, 2010). This implies that mutuality and participation occur in the encounter between professionals and parents. Nevertheless, research with children shows that they want to participate in their health care situations and in planning their own treatment (Oldfield & Fowler, 2004). Additional research involving children shows that they have not been given the desired amount of information and not been listened to in their encounters with professionals (Coad & Shaw, 2008; Coyne, 2006a, 2006b, 2008; Coyne, Hayes, & Gallagher, 2009; Jolley, 2006; Lambert, Glacken, & McCarron, 2008; Lambert et al., 2010; Oldfield & Fowler, 2004; Söderbäck, 2012). Other studies show that children feel a lack of support and that they have not been given the opportunity to participate in their own care; and that their perspective has not been taken seriously (Coad & Shaw, 2008; Coyne, 2006a, 2006b, 2008; Coyne et al., 2009; Jolley, 2006; Lambert et al., 2008, 2010).

As already stated, health care situations with children always imply that the professionals encounter the child and his/her parent or guardian (Söderbäck et al., 2011). This study focuses on the complexity of such encounters. The significance of participation in these complex encounters needs to be described and clarified to promote children’s participation further. The aim of this study is to describe health care professionals’ understanding of significant encounters with children and parents to uncover the meaning of participation.

## Method

This study is a part of a project with the purpose to promote children’s participation in their health care situations (IACTA, 2012). This part of the project focuses the health care professionals understanding of significant encounters with children and parents to uncover the meaning of participation. The project lasted for one and a half years, from September 2014 until June 2016.

The approach in this empirical study is from the perspective of caring science, a human science aiming at supporting and strengthening human health. Such caring actions requires trustworthy encounters and it is necessary to identify individual’s experiences in a specific situation (i.e., Watson, 2008; Dahlberg & Segesten, 2010; Arman, Dahlberg, & Ekebergh, 2105). Methodologically, the study is in accordance with the phenomenological hermeneutical approach developed by Lindseth and Norberg (2004). Such approach hold that “taken for grantedness” needs to be reflected to become uncovered. The reality shows itself as understanding and meaning, and due to Gadamer (1989) this process is both phenomenological and hermeneutical. In this study, professionals’ narrated experiences of significant encounters with children are reflected to uncover the meaning of participation (Ricoeur, 1976).

### Participants and data collection

The professionals on a primary health care unit (n = 6) and a day ward unit for children in a regional hospital (n = 6) were invited take part in the study. The invited professionals encompasses registered nurses, physicians, assistant nurses and bio-medical analysis. The professionals experiences of encountering children and parents varied in the range of 2–15 years which imply they were familiar with the phenomenon of the study. Furthermore, the researchers had prolonged engagement with the informants in terms of monthly reflective forums during a period of 1½ years. Therefore, a trustful relation with the professionals was established which enabled a prolonged data collection period contributing to thick and rich data. The 12 professionals were encouraged to write narratives of their experienced significant encounters with children in health care situations. . The variations of professions represented in the study provides for a maximum variation sample. Their narratives (n = 35) constitute the data for this study.

The narratives varied from half a written page till two pages and the health care professionals wrote on average three narratives each. The narratives were written from the initial question: describe a significant encounter with younger children in your clinical work

### Analysis

Data analysis was conducted with a phenomenological-hermeneutic approach (Lindseth & Norberg, 2004). The purpose of this approach is to uncover the meaning of lived experiences through interpretation of narratives. The approach requires that data are analysed for patterns of meaning. According to Ricoeur (1976) experience is private but its meaning public, – an experience cannot be transferred to another person but what is transferred is the *meaning* of experiences. Interpretation of the texts constitutes to progress from understanding to explanation and from explanation to comprehension. This process is referred by Ricoeur (1976) as *mimesis*; – where understanding departs from what he names a pre-figurative state (naïve understanding) and on to the con-figurative state (critical analysis) and further into what he names as a re-figurative state (comprehensive understanding).

In this study the health care professionals’ narratives were analyzed by the three authors in cooperation with an intentional bridling of preconceptions and reflexive awareness. The authors have specialized training as Paediatric nurse (MS) and as Public Health Nurse (AR, MH) such experiences include having own experiences of encountering children in child health care. The pre-understanding of encountering children were helpful in the interpretation of the narratives. The analysis of the narratives involved three methodological stages. In the first dialectic phase a naïve reading of the narratives as a whole to gain an initial impression was performed. The texts were read several times with an open attitude, allowing the text to affect and speak to the reader. This phase represents the phenomenological approach to the text. The approach necessitates setting aside one’s pre-conceptions to reflect on what is taken for granted- and become capable of surprise. As such, a primary understanding of the phenomenon is gained (Lindseth & Norberg, 2004). Questions emerged in the naïve reading guided the next phase of the analysis: the thematic structural analysis. In this phase all the narratives were unpacked of meaning. Each narrative represented meaning units and they were condensed to uncover the professionals’ understanding of their significant encounters with children and parents from the perspective of participation. The condensations were discussed in relation to variations and similarities and abstracted into subthemes and further to the main theme: Participation as movement built up by participation characterized as both happening in mutuality as well as in alienation.

The analysis was validated by the authors through elaborating and discussing the findings in relation to the first naïve understanding. Finally, in the last phase a comprehensive understanding was constructed to create an in depth interpretation. The comprehensive understanding was first based on the naïve understanding, the structural analysis, the researchers’ pre-understanding and then related to Buber’s philosophy (1994) of the I-Thou relation. At all stages the analysis process was conducted by the three researchers; – discussed, triangulated and processed to expand the understanding of the phenomenon in focus (Morse, 2015) i.e., health care professionals’ understanding of significant encounters with children and parents to uncover the meaning of participation.

The comprehensive understanding revealed new possibilities for being in the prefigured world of the health personal as described in the narratives, and refigured in the researcher’s interpretation and in the interpretation of the readers of the research paper (Ricoeur, 1976).

### Ethical considerations

The ethical principles from the World Medical Association (2009) were applied when conducting the study. Each professional was given written and oral information about the aim of the study, and that data were to be treated confidentially (SFS, 1998:204) and that they had the right to withdraw from the study at any time. The health care professionals gave their written informed consent to take part in the study prior to the data collection. According to Swedish law (SFS, 2003:460), the approval of an official research ethics committee is not required for this kind of research as no children and parents were involved.

## Findings

### Participation; encounters in mutuality or alienation

In health care situations, the professional, the child and the parent can participate in mutuality or in alienation. Mutuality in a health care situation means that the action; examination or treatment, is carried out by the health care professional together with the child based on mutual respect and understanding. They are participating in the situation. Alienation in such situations means they do not actually encounter each other; their individual intentionality is directed elsewhere. As they do not establish a relationship, the respect and understanding of one another can be compromised. The persons participate but are not participating.

#### To participate in mutuality

A health care situation in which the actors participate in mutuality means that they encounter each other as a three-part unit and they create a relation. They contribute their perspectives in the situation and adjust to each other’s understandings of the purpose of the situation. In such health care situations, the parent provides the child with bodily or verbal support and lets the child form a relationship with the professional. The professional is pliable towards the child and recognizes the child’s state of being. When acting in mutuality the professionals create conditions that promote participation. They provide space for the child to tune in to the situation. They inform about how the examination will be done, and offer a new appointment if the child does not want to, or does not have the energy to continue the examination. Furthermore, the professional may set up the terms in the situation but in such a way that the child can accept them. The situation occurs from the basis of the child’s terms i.e., the child’s experiences, competence and emotional state, which also provide space for the child’s possibility to take initiatives of his or her own and have control. The child is invited to show and tell about his/her treatment and how it needs to be done so he/she can feel secure and be able to master the situation with his/her own strategies. When the child is worried or difficult to reach, the professionals, by being confident, can negotiate and guide the child through the situation. They can also reach the child by being patient, trying different courses of action, or taking the lead and thereby helping the child to master the situation. In such situations the professionals need to keep their promises to not alienate the child. The following meaningful narrative describes a situation in which the persons involved participate in mutuality.

Today, a five-year-old girl came to learn how to breathe in a spacer without a mask and to blow in the spirometer. Blowing into a spirometer at such a young age usually does not work so well, but I had promised the doctor to make an effort. We began practicing with the spacer without a mask and I told her it’s like blowing out candles on a cake. The girl then lit up because she had recently celebrated her fifth birthday, and the birthday cake had candles. She managed the spacer without a mask very well, and then it was time for the spirometer which requires technique and the courage to take a deep breath and exhale hard. The girl could hardly reach up to the table where the computer stood and the mouthpiece on the spirometer was large for her mouth. I explained the procedure to her; that she needed to help the fireman on the computer screen to put out the fire by blowing as hard as she could into the mouthpiece. She put the mouthpiece in her mouth, breathed in and then blew out as hard as she could. She followed the instructions exactly and the fire went out. I am surprised it worked so well, I had a pre-conceived idea how it would go given her age but it worked out really well.

#### To participate in alienation

In health care situations the child, parent and the professional can participate but without actually encountering each other as a three-part unit. They do not adjust to each other’s understandings of the purpose of the situation or are not clear about the consensual expectations. Some of those involved may adjust to each other but some are alienated, or alienate themselves, from the unit.

To participate in alienation can be given significance in various ways in health care situations. In some situation the professional and the child are directed towards one another but the parent is directed towards him-herself. The professional tries to promote the child’s understanding and participation in the situation by informing and showing what will happen. The child adjusts and goes through with the examination but simultaneously the parent takes a superior position by using a language only she understands instead of supporting the child. Parents can also take a superior position over their children by limiting their opportunity to encounter the professionals themselves. This occurs when parents report their child in the reception and leave the child outside or talk on the child’s behalf.

In other health care situations, the professional and parent are directed towards each other e.g., when they want the child to cooperate in an examination or when the professional want to create mutuality in understanding in the three-part unit. The child on the other hand protests or gets sad, and alienates him-/herself. This may be due to too many persons being involved, the individuals’ emotional state, the prevailing atmosphere, or how confident the professionals and the parents are. Furthermore, the children’s alienation may have to do with their wish to not participate or their competence to understand the situation. However, when the situation is ended the child may turn towards the professional, making eye contact, smiling and raising a hand in greeting.

In further other situations, the persons involved do not reach a state where a relation is established. In these situations, the professional are directed towards the child by invitations, providing information about what will happen and what they are doing, and giving instructions. Also, the parents can be given instructions so they can support the child and facilitate the situation. However, the professionals are not encountered by the attention they expect. The parents may act sceptically towards the professional. Regarding the child, the parent can ignore or laugh at the child’s bad acting, or may have neglected to tell the child about the examination or treatment before meeting the professional. This does not facilitate the situation, and the child does not agree to go through with the examinations. In these situations, the professionals can sense pressure or a strained atmosphere, lack of confidence from the parent but also the professionals may feel angry or frustrated. When the professionals invite the child to an examination or are trying to carry out an examination they are ignored, shown bodily anger or screamed at in fear, and the parents neither restrict nor try to calm the child. Even if the professionals try to calm the child and the child listens and tries to adjust, he or she fails to do so. This failure to carry through an examination may be due to the lack of parental support, the child´s sense of the parents’ scepticism towards the professionals, or lack of time and space for the child to get prepared. In situations in which the child has not yet developed the verbal and cognitive competence needed to explain his/her difficulties and the parent does not have knowledge about these difficulties, the professionals want to help the child but cannot.

The following meaningful narrative shows an encounter in which the professional, the parent and the child are alienated towards each other.

On my way to the coffee break, I saw a mother who took off her outer clothes in the waiting room but the child, a four-year-old girl had her outer clothes on. There was also a baby boy in a pushchair. During the coffee break, I heard the girl scream and thought: “I hope that is not the child I will soon meet”. On the way back from the coffee break, I saw the family again. The mother was taking the girl’s outer clothes off. The girl was quite dejected and I understood the situation as that she was “overheated”. I remembered I was feeling good, wasn’t stressed and felt the anticipation of the visit. I called the girl’s name and the mother brought the pushchair and entered the consultation room without the girl. The girl played in the waiting room. In order to attract attention, the girl holds mum’s cell phone in one hand and had her own toy phone in the other hand. It was clearly the girl who determined the conditions and she did not want to meet any doctor, at least not for the moment. The baby boy was sleeping in his pushchair, but still the mother concentrated on him and seemed to think it was my job to get the girl from the waiting room. I tried to show interest in her phones and sat next to her. She was not interested in contact but ran to her mother. I asked the mother to sit down and take the girl into her lap. I usually ask the child first if they know why they have come, but now I turned directly to the mother, who told me about the girl’s stomach problem. When I asked counter questions about when, how long and in what situations the problem occurred she could not answer. She did not know anything about the girl’s faeces consistency or frequency even though she helped the girl after toileting. I interpreted this to mean she had not thought that much about the girl’s stomach problem. I thought that the problems had begun in connection with the little brother’s birth, but the mother strongly denied that. It was quite impossible to conduct a bodily examination and when I tried to get the mother to accept that we needed to work together, she focused on the baby sleeping in the pushchair. It seemed as if the family solves problems by “flight”. The girl by playing with the phones and wriggling out of her mother’s grip, and the mother by focusing on the baby when the girl needed attention. This could have kept on indefinitely, but I made the decision to do the opposite. The examination was impossible to accomplish but there was nothing alarming about the girl’s condition. I explained to the mother that she had to prepare the girl for the visit, encourage her child to be “good” and to not have distractions at hand and then we could set up another appointment. I told the mother to think about the questions I had asked, I gave some advice concerning the regularity of eating habits and then I opened the door and said “goodbye” and that they were welcome back. This took 15 minutes and the mother looked mightily surprised.

#### Participation as movement

Participation is dynamic in its nature, meaning it can vary within a health care situation or between different situations involving the same persons. How this movement appears has to do with each person’s actions in the situation. This movement continuously occur in mutual understanding and harmony, as when the individuals are directed towards each other. They adjust to each other to make the situation proceed, meaning that the examination or treatment is conducted in mutuality. This movement may change from being proceeded in mutuality to become alienated, or the other way around. Participation always occurs in encounters with others, and varies depending on the persons. Therefore, participation can be understood as being situated. The following two meaningful narratives show participation as movement. The first narrative shows participation as movement within the same situation.

A seven-year-old girl came with her parents. In the consulting room was my chair and two more. The parents placed themselves in the chairs, and the girl sat on the examination bed. She looked very tense so I asked her if she found the situation frightening. She never got to answer, her mum confirmed that the girl was very nervous and had talked about the visit for days. Then I went through what I was going to do and how to do it, and told her that if we helped each other this would work out fine. She got to choose in which order the different examinations should proceed. It worked out well, but she was very tense. When the examination was over, I told her she had been brave and now the examinations were finished. Then I summarized the results and made the judgment that her height and weight were needed. This, became too much for the girl and she started to cry. The measurements were taken but it was obvious she was embarrassed by her own crying.

The following, second narrative, shows the movement as it can occur between two different situations with the same persons involved.

A four-year-old boy came for a health visit who did not want to cooperate. As I read out the weight scale I noticed that he had climbed two divergences up on the scale, and we made up another appointment for a follow up within the next six months. By the time of the next visit I went out to meet with met the boy in the waiting hall. He was very forward and cooperated. When he put his clothes back on after the weight check, he approached me cheerfully asking for help with his shoes and said: I like you!

### The comprehensive understanding

Encounters in health care situations between a professional and a child can be understood as an I-Thou relation (Buber, 1994) characterized by participation as a movement between mutuality and alienation. The I-Thou-relation may enable children to participate and experience themselves as persons with opportunities. However, health care situations with children always implies encountering their parents or guardians as well (Söderbäck et al., 2011). The encounter is an I-Thou relation but there will be one I and at least two Thous. In an I-Thou relation, aspects of mutuality is essential (Buber, 1994). In an encounter, the mutuality between the I and Thou is always limited by the involved persons’ understandings of each other and the situation. This limitation may influence the movement of participation in mutuality towards participation in alienation. Being aware of this limitation facilitates the understanding of participation as movement. The child and the parent are the ones seeking care, the health care professionals are the ones knowing about and conducting the examinations and treatments needed. The acceptance of each other as persons with varying wishes, thoughts, abilities experience and knowledge is therefore significant (Buber, 1994). This is related to the child being confirmed as a person, including trying to see each child’s developmental skills and opportunities from the child’s perspective (Sommer et al., 2010). As in the narrative with the 7-year-old girl being very tense; the professional gives the girl information and an opportunity to choose the examination order, and confirms her courage. The girl goes through with the examinations needed even though she did not know about them from the beginning and even though she cries. The asymmetry between the professional and the child is obvious, as well as the acceptance and promotion of the child’s participation on her own terms as stated in the Convention on the Rights of the Child (United Nations, 1989).

A limitation regarding the prerequisites for mutuality to occur is that both the I and Thou in the relation depend on the child’s age and development. The child may have limited abilities to express needs, wishes and thoughts. The professional on the other hand may have difficulties in understanding the child’s expressions. The parents and professional may strive to do what is best for the child as in accordance to the Convention on the Rights of the Child (United Nations, 1989) and The Patient Act (SFS, 2014: 821). Still, they may act against the child’s wishes and needs. The relation may end up in an I-It relation (Buber, 1994) in which participation occurs in alienation. Consequently, these persons participate but are not participating. The child is overseen, and only the occasion for seeking care is taken into account. In such situations the parent and the health care professional are directed towards each other. It is possible that the child wants to alienate him/her self from the encounter and prefers the I-It relation as a way to master the situation, for example when there are too many people involved for the child to deal with.

The professionals’ need to recognize the child’s skills and concerns as they otherwise will be unknown and unconsidered. This requires conditions such as open awareness, time, energy and patience. Smythe, Payne, Wilson, Paddy, and Heard (2014) say professionals need to act tactfully, meaning they need to be engaged and open to what is happening in a situation. They must also be able to listen, observe and understand the child to achieve mutuality.

This is seen in this study for example in the narrative of the 5-year-old girl accomplishing an examination including a spacer and a spirometer. The professional has preconceptions about the expected difficulties in the situation but also recognizes the girl’s experiences and skills to participate in mutuality. The professional’s encounter with the girl tactfully in a mutual I-Thou relation. In exchange, the professional gets an additional perspective on what a child is able to deal with despite age and bodily maturity. As mutuality and participation occurs as a movement it involves the ability to be directed towards each other as in an I-Thou-relation in a situation (Buber, 1994). In the narratives used to illustrate the findings of this study it is possible to grasp the health care professionals’ efforts to encounter the child and their parents to reach participation in mutuality. However, the findings also show a professional’s decision to not encounter the child nor the parent, as in the narrative with the 4-year-old girl with stomach problems in which the parent shows little interest in the situation. When the participating persons do not recognize each other in mutuality they participate in alienation which is not in line with the Convention on the Rights of the Child (United Nations, 1989) or The Patient Act (SFS, 2014: 821).

Mutuality among the persons in an encounter is essential and brought to light by their desires to participate and adjust to each other. Consequently, I-Thou relations are closely connected to the person’s experiences of meaningfulness in the encounter. Meaningfulness in health care situations means that the care provided is adjusted appropriately to those in need of it (Jordan et al., 2012).

### Methodological considerations

From a hermeneutical philosophical position caregivers continuously need to reflect upon situations and place themselves “in open awareness regardless of ontological connection or caregiving intention. This may contribute to broadening perspectives in their encounters with those seeking care. In the present study, the health care professionals placed themselves” in the open’ by writing narratives about significant encounters with children and their parents. These narratives illuminate the complex meaning of participation as a movement between mutuality and alienation, and that the individual’s intentions and willingness to act intersubjective influence the direction of this movement.

Hopefully, the analysis and the chosen narratives exemplify the meaning of participation as movement between mutuality and or alienation, but in the end it is up to the reader to consider the applicability and the soundness of the arguments being made.

In the analysis process, the three authors conducted the analysis individually and continuously held joint interpretative dialogues on the data in order to achieve trustworthiness. Questions were asked towards the narratives; putting them at play mirrored with theories as well as reflecting on them from a child’s perspective (Harder, Christensson, Coyne, & Söderbäck, 2011). Debriefing of the findings were presented to the professionals for reflections to secure inter-rater reliability and correction of researcher bias. The result of the analysis was also peer-reviewed by research colleagues. The authors intentions of challenging the movement between pre-conceived thoughts and practice is to expand awareness of what consequences actions have, both on patients, on colleagues and on the milieu; for healthcare professionals as well as for the researchers. An assumption is that in the effort of challenging the meaning and significance of our intentions, of why we think and act as we do, we are constantly deepening and clarifying our caring actions. The authors have strived to apply the method coherently and rigorously throughout the research process.

### Conclusion and implications for practice

Our findings suggest that participation in health care situations with children can be understood as movement in mutuality and alienation. Participation can continuously move between mutuality and alienation. Such understanding is useful to support children’s opportunities to participate from their own perspective and their own opportunities to deal with and master necessary health care examinations in according to the Convention on the Rights of the Child (United Nations, 1989) or The Patient Act (SFS, 2014: 821). The understanding of participation as movement require professionals to be aware of that their understandings of a situation may differ from those of the child and/or the parents. Such understanding also requires a consciously strive for a meaningful I-Thou relation. This is possible when individual adjustments are found in joint actions in the three-part unit, professional-child-parent/s. Clinical reasoning in health care situations with children involves manipulation of the environment, and artefacts as well as hands-on practice. Furthermore, clinical reasoning involves reflective questions such as: What actions do I use in a situation with a child? How do I act towards a child? Why do I act the way I do? For whom do I act the way I do? Neither the progression nor the outcome of a health care situation can be predicted. Still, professionals can be aware of their values, preconceptions and wishes that direct their encounters with children and their parents.
